# Health care seeking and financial behaviours of the elderly during wartime in Goma, Democratic Republic of Congo

**DOI:** 10.4102/phcfm.v2i1.108

**Published:** 2010-06-14

**Authors:** M. Prosper Lutala, Timothée M. Kwalya, Eric K. Kasagila, L. Hubert Watongoka, Bavon W. Mupenda

**Affiliations:** 1 Department of Family Medicine, University of Goma, DRC; 2 Chercheur Indépendant Programme Elargi des Vaccinations, DRC; 3 Department of Community Health School, Public Health College of Medicine, Malawi; 4 Chercheur Indépendant Programme Ecole Sans Enseignant Bukavu, DRC; 5 Centre Interdisciplinaire de Bioéthique pour L'Afrique Francophone, DRC

**Keywords:** Congo, the elderly, health seeking, post-conflict geriatric care, financial behaviours

## Abstract

**Background:**

Health and social services utilisation is seen to be more closely related to age than to other socio-demographic characteristics. Many health problems are known to increase with age and this demographic trend may lead to an increase in the absolute number of health conditions in this population. However, questions are still emerging as to how the elderly seek care in response to their needs in the context of a war-torn region.

**Objectives:**

The aim of this study was to determine the behaviour of the elderly in seeking care during a time of conflict.

**Method:**

A descriptive cross-sectional study was carried out in the health district Goma, in the Democratic Republic of the Congo (DRC), using a multistage sampling of 500 senior citizens. Eight trained field-workers were deployed in the field where they administered a structured questionnaire.

**Results:**

The public health sector was well known and preferred by 186 participants (37.2%), but only used by 16 (3.2%) participants. Financial support received by the elderly came from their own relatives and fellow believers in 33.5% and 20.2% of cases, respectively. Almost 71% of monetary support is the result of begging and unknown sources – there is no government involvement whatsoever. Much of the external support that the elderly receive involves support in the form of food. Disease expenses remain a main concern of the elderly themselves.

**Conclusion:**

Government support for the elderly in the DRC is non-existent. There is an overuse of private sector and traditional medicine, despite the preference indicated for the public health sector. As a recommendation, a general increase in income-related activities could contribute to alleviating the health state of the elderly in a war situation. Further studies might explore in future the contribution of those results on the health of elders.

## INTRODUCTION

Health and social services utilisation is seen to be more closely related to age than to other socio-demographic characteristics. Many health problems are known to increase with age and this demographic trend may lead to an increase in the total number of health conditions in this population.^1^ In addition, because there is a growing body of evidence that older people are at risk of multiple co-morbid conditions, their search for health care will probably also increase. This is especially important when characterising older adults, who are the group most at risk where disease, disability, a dependent life and requiring costly health care services are concerned.^2^ The attribution of ill health to ageing, low economic status and a negative attitude of health workers toward the care of the elderly are some of the factors associated with a delay in seeking health care.^3–5^

The number of senior citizens aged 60 years and older is increasing rapidly worldwide. The world's population aged 80 years and older is projected to increase by 233% between 2008 and 2040, compared with 160% for the population aged 65 and older and 33% for the total population of all ages.^6^ The worldwide population of elderly people was estimated to be 20 million in 1959; by 1975 it had increased by 75%, reaching 600 million.^7^ Today, the African elderly population is estimated to be 38 million and is projected to reach 212 million by the 2050s. In some African countries, such as Malawi, South Africa, Zambia and Zimbabwe, where HIV/AIDS is particularly devastating, the average life expectancy at birth is less than 45 years^6^, while the population of the elderly continues to increase.^8^

In sub-Saharan African countries, the level of assistance from community members is known to be very high. This has contributed to some vulnerable people being assisted in coping with various threats, including health problems. However, increasing urbanisation and emphasis on the nuclear family and individualism have caused many people, and also some African governments, to fear that the care-taking family system may no longer be the support system that one would like it to be.^9^ In addition, at state level, there are many factors contributing to increasing this fear, such as armed conflicts, mismanagement of state's assets, impact of International Monetary Fund policies on governments and fees for service as a mode of payment. Consequently, most African health systems are affected and are failing to cope with the health requirements of the population, particularly those of the most vulnerable.

It is also known that the major elements of health status are mediated by the quality of care provided to the population. This quality seems very important in various vulnerable populations, such as the under-fives, pregnant women, people affected by chronic diseases (e.g. tuberculosis and HIV) and the elderly.

According to some anecdotal evidence, the population of Goma is unable to cope with diseases or any threat due to a high vulnerability. If the younger population is able to overcome some or all such diseases and threats, it would be interesting to know how the elderly are coping in the same environment.

The first step is thus to identify some of the elderly's common problems, their health care seeking behaviour, as well as other challenges they encounter. Therefore, we conducted this study, which was part of a larger survey, to identify the ways in which the elderly, during a time of conflict, seek health care (within the entire health care system). The specific objectives of this study were:

- To determine knowledge of, the acceptability level among and utilisation of formal health care systems by the elderly.
- To identify structures used by the elderly in the case of diseases.
- To identify sources of money used to cover the cost of this health care.
- To identify social support systems used to access such care.

## METHOD

This is a descriptive cross-sectional study. It was carried out between the beginning of April and 20 May 2003, in an urban– rural health zone of Goma, in the eastern DRC. The town of Goma is located about 2000 km from Kinshasa, the capital city of the DRC. It is characterised by chronic crises, including interethnic conflicts and civil wars. Furthermore, in January 2002, Goma was affected by a volcanic eruption that destroyed two thirds of the economic sources of the town.

In this study we used a combination of sampling methods. The subject was a population who had been living in Goma for five years prior to the volcanic eruption. We commenced with maximum variation sampling, with North/South and rural/ urban combination criteria, to select the sites.

From a list of 20 catchments areas, four were selected based on geographical location (the extreme north and south of the city) and remoteness level (rural and urban). These areas were: Kibumba and Kanyarutshinya (in the sub-rural North) and Mabanga and Charité Maternelle (in the urban South). After the pilot study, Charité Maternelle was removed from the selection list due to lack of involvement of the stakeholders from this catchment area. Cluster sampling was then done in suburban and urban settings, respectively, for selecting villages and streets. After dividing the selected areas into villages (in Kanyarutshinya, Bujovu) and streets (in Mabanga) a simple random selection of three villages (for sub-rural areas) and three streets (for urban areas) was carried out. In the areas selected above, all households with elderly people fitting our definition of the elderly (see below) were included until we reached our sample size.

A sample size of 500 elderly people was selected in the six areas. As no such study had ever been conducted here, and no systematic list of the elderly was available, the number of 500 households was chosen purposively, bearing in mind that a sample size of 500 people can be considered adequate to generate reliable results. For the purpose of this study, the elderly were defined as people of both sexes aged 60 years or older.^8^

A training session of about 7 h per day, for 2 days, was held for the field-workers. The content of the training was directed at teaching them some of the basics of research methodology, how to conduct interviews, the content of the questionnaire to be used in this study, how the questionnaire should be completed and some administrative tasks related to the process. The questionnaire is shown in Box 1. Prior to data collection, a pilot stage, followed by reformulation of the questionnaire, was held in Bujovu and Casop (two non-selected catchment areas in the study). Eight field-workers were deployed to collect data.

Box 1 Questionnaire used during data collection for the elderly in Goma

Access to health services has become very complicated. It is even more complicated for elderly persons who are weak, as well as for children, orphans and so on. But each person has his own way of experiencing the situation on the ground, so we decided to find out more about the ways in which elderly people deal with their health and the support they receive from external sources. This is why we are requesting that you respond to some questions that would take an average of 30 minutes to answer. This study was launched by DOCS and one NGO, HelpAge International, who is planning to implement some activities that target the elderly.

1. What is the first action taken once you get too sick to access care?
2. What is (are) reason(s) for choosing where the treatment is sought when you are sick?
3. What is your age? Marital status? Sex?
4. As a man/woman over the age of 60 years, do you know the types of structures that can take care of you or fellow elderly people once you are sick? If yes, can you please provide their name/s?
5. Can you tell me about the attitude of health providers when you go to a health facility? In other words, what kind of welcome are you receiving from health providers once you go for consultation?
6. Who supports you to cover the costs of treatment/care expenses when you are sick?
7. Once they support you, can you tell us what nature of support are you receive from the above sources to cover your treatment/care?
8. Despite any support received in the form of food, clothes, etc., you would still need cash to cover your basics needs, as well as medical costs. Since you are no longer active in the work environment, can you tell us from which sources do you get such money?
9. What is your order of preference for the health structures you like to attend once you get sick, if you are given money to cover all treatment costs?
10. Do you have any further comments related to our topic?

Thank you very much for taking your time to answer to our questions.

Interviews, using structured questionnaires, were administered either in Kiswahili and/or Kinyabwisha. They included demographic information, and questions about reported diseases and health behaviour.

Supervision was conducted on a daily basis by a team of two supervisors, whose task was to discuss any difficulties encountered during data collection and to find appropriate solutions. All data were captured on Microsoft Excel by a team of three assistant researchers, who were computer literate students, skilled in the public health field.

Authorisation to conduct the study was sought and obtained from the provincial inspector of the health office, the health district manager and street/village headman of the four selected catchment areas. Individual consent, after an explanation, was sought a day prior to the interview in the presence of a young, literate relative and an oral reminder was obtained before commencing with the interview the following day.

## RESULTS

### Socio-demographic data

Five hundred elderly people were admitted to the study: 234 (46.8%) were between 60 and 70 years old, 250 (50%) were between 71 and 80 years old and 16 (3.2%) were above 80 years old. There were more men than women (350 men and 150 women; 70% and 30%, respectively). Most participants were widowed 218 (43.6%), 200 were married (40%) and 32 (6.2%) were separated. These percentages were rounded up in the graphs.

### Knowledge and attitude towards health structure by elders

The modern health structure was known by 186 (37.2%) participants, 32 (6.4%) stated they were unaware of any health facility and 282 (57%) were unsure of an answer to this question.

### Sources and nature of support

**FIGURE 1:**
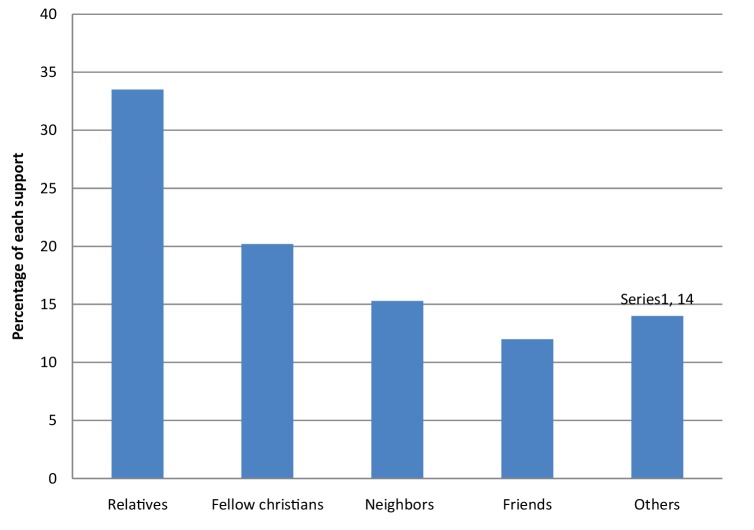
Sources of support for the elderly in Goma

Figure 1 shows that relatives and fellow Christians were found to be the main sources of support for the elderly in Goma for 106 (33.5%) and 64 (20.2%) of participants, respectively.

**FIGURE 2:**
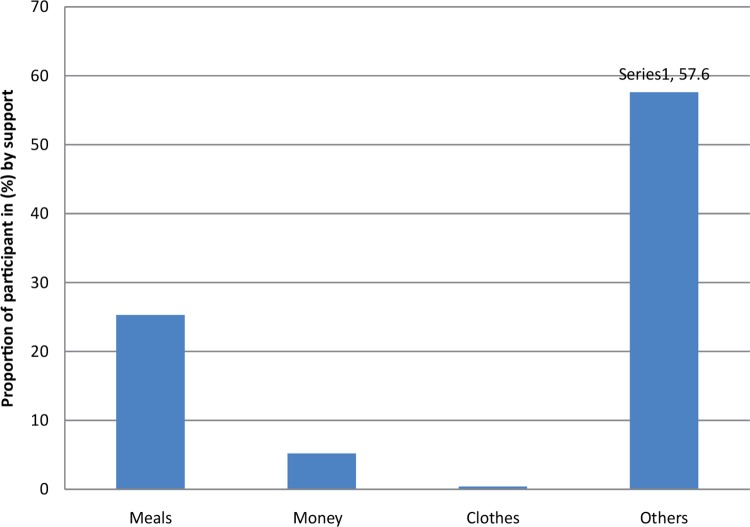
Nature of support for the elderly in Goma

In terms of the nature of help (Figure 2), meals were found to be the main source for 80 participants (25.3%), followed by money for 26 (5.2%). Upon investigating other sources of support, it was found that the elderly received assistance in various ways: they were given firewood and water for cooking, they were visited (a sign of assistance) and they were assisted in the area of farming, for example, once the harvest was ready, other people sold the harvest products for the elderly. In other circumstances, s ome people offered to escort the elderly to the church on Sundays, while others assisted them with writing letters to send to relatives; others escorted them when taking a bath, had discussions with them when they were lonely and helped to treat their body rashes.

**FIGURE 3:**
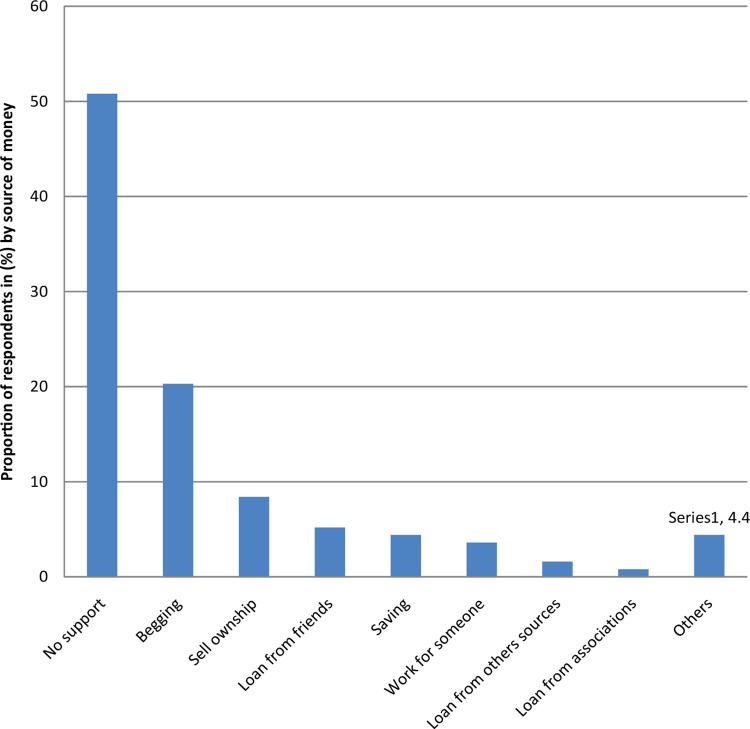
Sources of money used to finance the care of the elderly in Goma

No formal support (no support and begging) represented almost three-quarters of the source of money used by the elderly to finance their care in Goma (71.1%, Figure 3). No support from government was mentioned as a source of financing by any of the respondents.

### Actions taken by the elderly once they were ill

**FIGURE 4:**
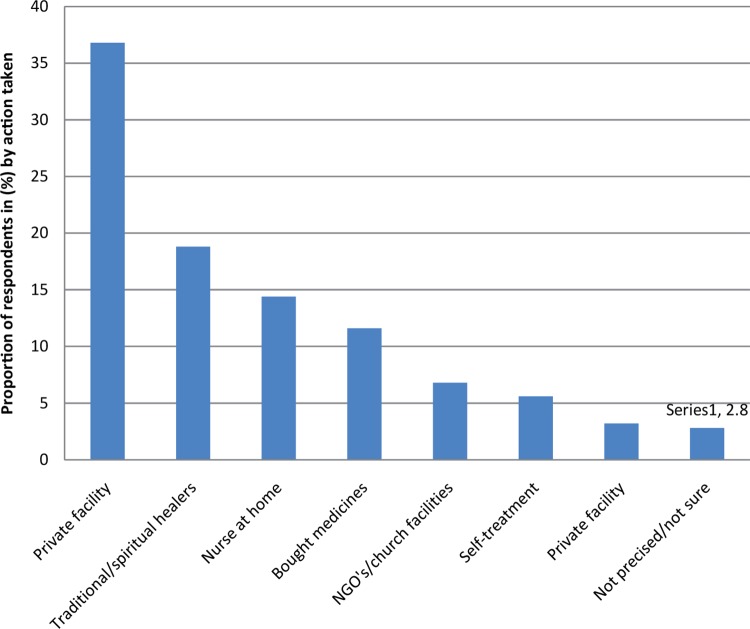
Action taken by the elderly of Goma once they were ill

Figure 4 shows what respondents reported as far as actions taken by the elderly themselves once they are ill. Private facilities and traditional spiritual healers were consulted by more than half the elderly once they were ill (55.6% of participants). The public health facility was used by only 3.3%.

### Facilities preferred by the elderly

**FIGURE 5:**
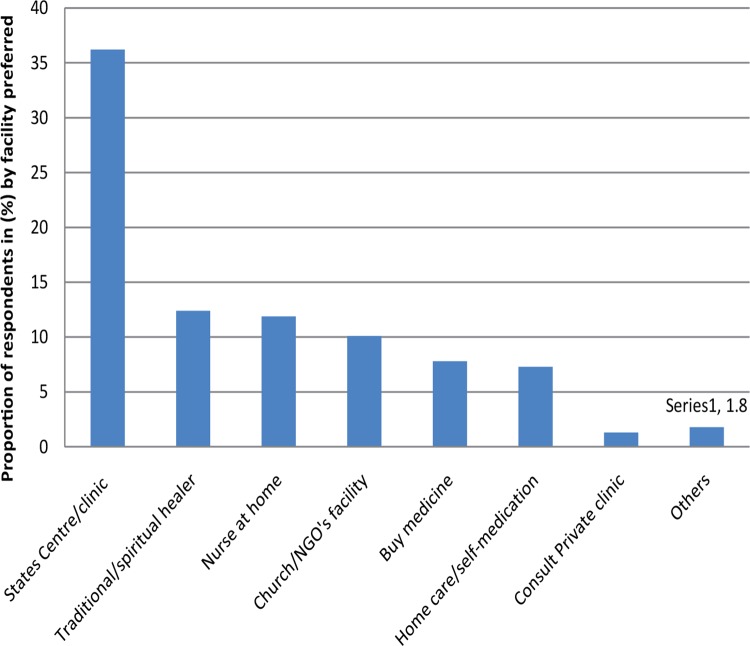
Facilities preferred by the elderly of Goma

We also investigated the facilities that the elderly preferred (Figure 5). More than a third of respondents stated that they preferred public health facilities; only 1.2% stated they preferred private health facilities.

## DISCUSSION

Most patients sought treatment from Western medicine and traditional healers. A combination of folk and modern medicine is not specific to Congo. Other studies conducted elsewhere in Africa led to the same conclusion.^10^ In many cases, traditional medicine appears to be complementing care, rather than substituting care provided by Western public and private doctors. There has been a marked decline in the quality of health services in countries affected by crisis. On-going economic difficulties have undermined the public health system, resulting in an increase in the ‘informal’ private sector, like traditional medicine.^11^

An opposite observation to this was made in Botswana,^12^ however, where traditional medicine was not used more often by the elderly. This can also be the case in Goma, if we extrapolate from the somewhat contradictory results we have found, namely an indicated preference for government facilities (public health services) but, in reality, we found an overuse of the private sector combined with traditional medicine. This finding may simply be a type of resilience mechanism, developed by the elderly, to cope with their needs in a health system that offers little support for vulnerable people. This complex pattern of health utilisation can also emerge from the under-reporting of health system use by the elderly, as previously described by Wallihan et al.^13^

The low quality of care provided by the public health sector to those who seek medical care can explain this. In fact, many clinics and hospitals belonging to the state in the DRC are ‘empty’: materials are lacking, there are staff shortages and those active staff work under very challenging conditions and are unmotivated. It currently appears that those lacking sufficient money to buy medicines and some emergency supplies prefer to go directly to where they can pay a fixed amount for a package of sufficient supplies for the duration of the illness.

In a system where fees are payable, without any clear social security scheme in place, people will experience difficulties when needing to access health care. The lack of health insurance has been identified as one of the major access barriers to health care.^14^ The introduction of fees was insufficient to boost the quality of health care, notwithstanding the assumption by the Bamako Initiative in Mali that the revenue generated by the introduction of fees for services in the health system would lead to an improvement in the health services by improving drug availability and, with the goal of improving quality of service, extend coverage and ensure equity in access to care.^11^ The impact of the policy on the access to care was actually very negative in some countries: there was a decline in attendance at health facilities in Ghana due to high costs, there was a 42% decline in the numbers presenting for health services in a fee-charging facility in Kenya and there was a 50% decline in the use of outpatient facilities in Tanzania after the introduction of user fees.^15^ Even if the figures are still unknown in the Goma health district, in Rwanda and the rest of the DRC, health facilities data showed higher utilisation rates for the insured than for the uninsured.^16, 17^ Also, with the quasi-absence of a social security scheme in the health district, we can easily understand how the elderly are not using the Western system. In Thailand, insurance aside, a substantial number of people continued to experience one or more serious difficulties when attempting to obtain much-needed medical services. These included financial, temporal, geographic and attitudinal difficulties.^18, 19^ These factors were not explored individually in the current study, but could also play a role in Goma.

We did not find any facility/medical department designed to specifically address the needs of the elderly. Barriers that impede the ability of families to obtain health care for aged members included, among others, a health care system that is not responsive to their unique needs and the problem of access to care if one or more family members are uninsured or unable to pay for services.^20^ Transportation, inadequate funds, inability to identify a provider, rude health care professionals and waiting time have also been identified as barriers to accessing health care. Further local studies could explore these determinants of service utilisation by the elderly.

The gender ratio of our sample can, to some extent, explain the low use of health services. In our study, gender was unequally distributed in the favouring of men. It has been established that men consult doctors less often than women do; women have a greater acceptance of seeking care.^21–30^

### Limitations

This study relied on maximum variation sampling and might, therefore, not necessarily provide data that are as accurate as could otherwise have been obtained from random sampling. Moreover, being a cross-sectional descriptive study, results simply provided a picture of the reality, whereas a prospective study would have yielded much more information on any changes that could have occurred over time. Nonetheless, the size of the sample was large enough to permit some reasonable generalisation to other elderly people's health seeking behaviour. The large difference between the preference of health facilities as indicated by the elderly and their actual use of the facilities, as found in this study, might lead us to thinking that there was a collector bias. However, our control of the data collection process enables us to conclude that this result is actually what the respondent gave the interviewers. A possible explanation for this is that proximity to private sector facilities might have caused the elderly people to use them, although they might have preferred going to a public health facility. Furthermore, due to the training of all field-workers, their strong background in research field-work and close monitoring on a daily basis by two supervisors, the likelihood of bias was minimal.

## CONCLUSION

Results of this study showed that, despite preferring the government's health facilities, many of the elderly in Goma consult either private health facilities or traditional healers. Their social capital is very strong, but weakened by several factors, including a lack of government involvement, the nature of support received from society and a weak health system. As a result, the elderly subsequently overuse the private sector and traditional medicine. An assessment of the predictors of this private use, the quality of care in the private and public facilities, barriers to access to care, as well as the correlation between income and use of different services by the elderly could shed more light in this under-explored field.

## References

[1] Gijsen R, Hoeymans N, Schellevis F, Ruwaard D, Satariano W, Van den Bos G. Causes and consequences of comorbidity: A review. J Clin Epidemiol. 2001;54:661–674. 11438406 10.1016/s0895-4356(00)00363-2

[2] Femia E, Zarit S, Johansson B. The disablement process in very late life: A study of the oldest-old in Sweden. J Gerontol Psych Sci. 2001;56B:12–13. 10.1093/geronb/56.1.p12 11192333

[3] Gjorup TH, Henrick LE, Strongard E. A study of the attitude of elderly people to physical symptoms. J Chron Dis. 1987;40:1095–1098. 3680467 10.1016/0021-9681(87)90076-2

[4] Galinksy D. Ten years’ experience teaching geriatric medicine Isr J Med Sci. 1985;21:49–53. 3997483

[5] Narayan D, Nyamwaya DA. Participatory poverty assessment study in Kenya Nairobi: UNICEF/ODA/ AMREF publication; 1995.

[6] Ninsella K, He W. An aging world: 2008. International population reports Washington DC: U.S. Department of Health and Human Services National Institutes of Health, National Institute on Aging, U.S. Department of Commerce, Economics and Statistics Administration, U.S. Census Bureau; 2009.

[7] US Bureau of the Census HelpAge International; 1999.

[8] HelpAge International Annual report. HelpAge International; 2002.

[9] Clausen F, Sandberg E, Ingstad B, Hjortdahl P. Morbidity and health care utilisation among elderly people in Mmankgodi village, Botswana. J Epidemiol Community. 2000;54(1):58–63. 10.1136/jech.54.1.58 PMC1731545 10692964

[10] Case A, Menendez A, Ardington C. Health seeking behaviour in northern KwaZulu-Natal Somkhele: Africa Centre for Health and Population Studies; April 2005.

[11] World Health Organization (WHO) Guidelines for implementing the Bamako Initiative. Proceedings of the Regional Committee for Africa, 38th session, AFR/RC38/18 Rev 1 Brazaville: WHO; 1988.

[12] Tlou SD, Sandberg E. The elderly and their use of the health care system In: Bruun FJ, Mugabe M, Coombes Y, editors. The situation of the elderly in Botswana. Oslo: Lobo Grafisk, 1994; p. 93–99.

[13] Wallihan DB, Stump TE, Callahan CM. Accuracy of self-reported services use and patterns of care among urban older adults. Med Care. 1999;37(7):662–670. 10424637 10.1097/00005650-199907000-00006

[14] Flores G, Abreu M, Olivar MA, Kastner B. Access barriers to health care for Latino children. Arc Pediatr Adolesc Med. 1998;152:1119–1125. 10.1001/archpedi.152.11.1119 9811291

[15] Asenso-Okyere WK. Financing health care in Ghana. World Health Forum. 1995;16:86–91. 7873037

[16] Schneider P, Schott W, Bhawalkar M, Nandakumar A, Diop F. Paying for HIV/AIDS services – Lessons from national health accounts and community-based health insurance in Rwanda 1998–1999 Geneva: UNAIDS; 2001.

[17] Criel B, Van der Stuyft P, Van Lerberghe W. The Bwamanda hospital insurance scheme: Effective for whom? A study of its impact on hospital utilization patterns. Soc Sci Med. 1999;48:897–911. 10192557 10.1016/s0277-9536(98)00391-8

[18] Bashshur RL, Homan RK, Smith DG. Beyond the uninsured: Problems in access to care. Med Care. 1994;32:409–419. 8182970 10.1097/00005650-199405000-00001

[19] Hayward RA, Shapiro MF, Freeman HE, Corey CR. Inequities in health services among insured Americans: Do working age adults have less access to medical care than the elderly? N Engl J Med. 1998;318:1507–1512. 3367961 10.1056/NEJM198806093182305

[20] Schantz S, Charron SA, Folden SL. Health seeking behavior of Haitian families for their school aged children. J Cult Divers. 2003;10(2):62–68. 14508927

[21] Tudiver F, Talbot Y. Why don't men seek help? Family physicians’ perspectives on help-seeking behaviour in men. J Fam Pract. 1999;48:47–52. 9934383

[22] Courtenay W. Constructions of masculinity and their influence on men's well-being: A theory of gender and health. Soc Sci Med. 2000;50:1385–1401. 10741575 10.1016/s0277-9536(99)00390-1

[23] Courtenay W. Behavioural factors associated with disease, injury, and death among men: Evidence and implications of prevention. J Men's Stud. 2000;9:81–142.

[24] Purcell H. Time to reverse the descent of man. Lancet 1995;346:240.

[25] Burkitt G. Strategies for dealing with men in general practice. Aust Fam Physician. 1999;28:773–774. 10495522

[26] Smith A, Mischewski A, Gifford S. ‘They just treat you as a number’: aspects of men's experience in a Melbourne sexual health service. Venereology. 1999; 12:15–19.

[27] Schofield T, Connell R, Walker L, Wood J, Butland D. Understanding men's health and illness: A gender-relations approach to policy, research, and practice. J Am Coll Health. 2000;48:247–256. 10863868 10.1080/07448480009596266

[28] Chapple A, Ziebland S. Prostate cancer: Embodied experience and perceptions of masculinity. Sociol Health Illness. 2002;24:820–841.

[29] Seymour-Smith S, Wetherell M, Phoenix A. ‘My wife ordered me to come!’: A discursive analysis of doctors’ and nurses’ accounts of men's use of general practitioners. *J Health Psychol*. 2002;7:253–267. 22114249 10.1177/1359105302007003220

[30] Lee C, Owens RG. Issues for a psychology of men's health. *J Health Psychol*. 2002;7:209–217. 22114245 10.1177/1359105302007003215

